# Stem Cell-Derived Exosomes in Autism Spectrum Disorder

**DOI:** 10.3390/ijerph17030944

**Published:** 2020-02-04

**Authors:** Nicola Alessio, Anna Lisa Brigida, Gianfranco Peluso, Nicola Antonucci, Umberto Galderisi, Dario Siniscalco

**Affiliations:** 1 Department of Experimental Medicine, Division of Molecular Biology, Biotechnology and Histology. University of Campania “Luigi Vanvitelli”, via S. Maria di Costantinopoli 16, 80138 Naples, Italy; nicola.alessio@yahoo.it (N.A.); umberto.galderisi@unicampania.it (U.G.); 2 Italian Group for Studying Autism-GISA, 25018 Brescia, Italy; brigida.annalisa@gmail.com; 3 Research Institute on Terrestrial Ecosystems (IRET), National Research Council of Italy, (CNR), via P. Castellino 111, 80131 Naples, Italy; gianfranco.peluso@cnr.it; 4 Biomedical Centre for Autism Research and Therapy, 70126 Bari, Italy; info@antonucci.eu; 5 Centre for Autism—La Forza del Silenzio, 81036 Caserta, Italy

**Keywords:** exosomes, stem cells, translational approach, neuroinflammation, autism spectrum disorder

## Abstract

Neurodevelopmental lifelong pathologies defined by problems with social interaction, communication capacity and presence of repetitive/stereotyped clusters of behavior and interests are grouped under the definition of autism spectrum disorder (ASD). ASD prevalence is still increasing, indicating the need to identify specific biomarkers and novel pharmacotherapies. Neuroinflammation and neuro-immune cross-talk dysregulation are specific hallmarks of ASD, offering the possibility of treating these disorders by stem cell therapy. Indeed, cellular strategies have been postulated, proposed and applied to ASD. However, less is known about the molecular action mechanisms of stem cells. As a possibility, the positive and restorative effects mediated by stem cells could be due to their paracrine activity, by which stem cells produce and release several ameliorative and anti-inflammatory molecules. Among the secreted complex tools, exosomes are sub-organelles, enriched by RNA and proteins, that provide cell-to-cell communication. Exosomes could be the mediators of many stem cell-associated therapeutic activities. This review article describes the potential role of exosomes in alleviating ASD symptoms.

## 1. Exosomes Characterization

In 1987, Johnstone et al. described the term exosome for the first time [1]. They were able to harvest, through centrifugation at 100,000× *g* for 90 min, the vesicles released by culturing sheep reticulocytes.

Presently, exosomes show great utility as diagnostic and therapeutic tools, but the lack of standardization in terminology is still an obstacle for their use in clinical trials [2]. Indeed, many different terms have been used and proposed as a name for these sub-particles, such as microparticles, ectosomes and shedding microvesicles. However, on the basis of their cellular origin, extracellular vesicles (EVs) can be broadly classified into (i) exosomes, (ii) microvesicles (MVs) and (iii) apoptotic bodies [3].

In 2018, the International Society for Extracellular Vesicles (ISEV) proposed the Minimal Information for Studies of Extracellular Vesicles (MISEV). The term extracellular vesicle (EV) refers to “particles naturally released from the cell that are delimited by a lipid bilayer and cannot replicate” [4]. Exosomes are therefore defined as small-size vesicles (30–100 nm) that are resident in eukaryotic compartments. Exosomes can participate in regulating several key cellular functions. They can alter the activity of the target cells, transferring DNA, RNA and proteins (these molecules are defined as “cargo” content) to other cells [3]. Differently, the term microvesicles refers to sub-particles of 100–1000 nm [3]. Exosomes originate from several cell types such as platelets, mesenchymal stromal/stem cells (MSCs) and tumoral cells [5,6] and are isolated from many physiological fluids: urine [7], sperm [8], cerebrospinal fluid [9] and plasma [10]. Despite their origin, they show a homogeneous density (1.13–1.19 g/mL in sucrose) [3].

Exosomes also show a specific set of proteins that indicate their cellular origin, together with a conserved group of proteins, which makes them easy to identify [11]. It is very useful to study, from a molecular point of view, cell–cell interaction, mechanisms of internalization, specific recipient cell selection and potential for their use as a drug delivery tool. They are able to merge and secrete their contents into recipient cells—that can also be very far from the exosome origin—and consequently could control biochemical pathways in the targeted cell [12]. For example, the “exosomal shuttle RNA” is an RNA that is moved from one cell to another and might potentially regulate protein synthesis in the recipient cell [13].

Interestingly, exosome formation and relative content could be regulated by molecular signals arising from the cell of origin. As demonstrated by Park et al., hypoxia-exposed tumor cells release exosomes with angiogenic and metastatic activity, indicating that tumor cells are able to adapt to a hypoxic microenvironment by releasing exosomes to enhance angiogenesis for a more favorable environment or to facilitate metastasis [14]. Interestingly, the rate of exosomal secretion and the relative content are state-specific when comparing healthy and pathological cells. The peculiarity of these differences under different conditions makes them particularly interesting, not only in molecular characterization, but also in the possibility of moderating and/or changing the cargo content in order to use them in regenerative medicine.

## 2. Stem Cell-Derived Exosomes as Tool for Cell-Free Therapy

In general, the term cell therapy refers to medical practices in which a patient is injected, grafted or implanted with cellular material; generally, this means living cells. In recent years, this definition has taken on a broader meaning. Currently, the term cell therapy refers to all cellular sources with biological activity that determine modifications in vitro or in vivo [15]. Since the 1990s, cell therapies have begun to be used as potential treatment for neurological diseases and neurodegenerative disorders.

Today, cell therapy is directed toward several clinical pathologies in different tissues and by different modes of cell transplantation. The actions by which the cells perform their functions are as follows: (i) As a stem cell or progenitor cell or through engraftment and differentiation processes to repair the damaged tissue. In these scenarios, the cells home and integrate into the site of damage, replacing the injured tissue and thus enhancing the improved function of the tissue [16,17]; (ii) Another mechanism is through the ability of cells to secrete paracrine or endocrine soluble factors and vesicles (extracellular vesicles). These biological components could help the self-healing of the tissue [18,19,20].

Among the EVs, the exosomes, or small bubbles, are released from cells, especially from several types of eukaryotic cell, into narrowing tissues or cells for intracellular signaling [21]. They act as shuttles to send nucleic acids and proteins to other cells, in this way, allowing cell-to-cell communication and transporting molecules among both close and distant cells. In general, these released proteins are important regulators of intracellular information.

Exosomes have shown valuable insights in terms of practical functionality. Indeed, Zangh et al. demonstrated that EVs released from neural stem cells (NSCs) in different regions of the nervous system were involved in several aspects of brain function [22]. For example, EVs released from NSCs in the hypothalamus were able to control the process of aging. They demonstrated that EV-treated mice showed reduction in age-related changes, in comparison with vehicle-treated mice exhibiting many age-related disorders [22]. This fact could be due to the fact that the exosomes can enter through the blood-brain barrier, stimulating neuronal differentiation and growth and suppressing inflammatory processes within the brain tissue [23].

The positive effect of exosomes is amply documented in several pathologies: Lai et al. in 2018 suggested that MSC-derived exosomes could improve graft versus host disease in mice through the inhibition of CD4 T cells [24]. In addition, the exosomes exhibit immune-modulatory properties by activating the regulatory T cells and blocking Th17 cells; Cui et al. in 2018 concluded that adipose-derived MSCs (ADMSCs) exosomes were able to rescue ischemic myocardium from ischemia/reperfusion damage by the action of their anti-apoptotic and pro-survival properties on cardiomyocytic cells. This healing mechanism can now be used in regenerative medicine [25]; Zou et al. in 2016 showed that exosomes exert positive effects on repair of ischemic reperfusion damage triggered by acute kidney injury (AKI) and could promote angiogenesis by vascular endothelial growth factor (VEGF) increase, through a HIF-1α independent mechanism [26].

Alternatively, the exosomes can initiate the pathological course of cancers by transferring specific harmful molecules. In this contest, these vesicles emerge as promising biomarkers. In addition, exosomes could potentially act as drug-delivery vehicles or cell-free vaccines, providing alternative approaches for anticancer management [27].

Exosomes could, therefore, help in regulating damaged processes within our bodies. Patients with chronic inflammation, autoimmune diseases and other chronic degenerative diseases may benefit from including exosomes in their treatment [28,29,30]. Currently, exosome therapy is also used to treat orthopedic injuries and for anti-aging management [31,32], indicating that exosomes are reaching a wider use in regenerative medicine. In recent years, accumulating evidence highlights their success in treating cardiovascular diseases (CDs), strokes and cancer.

Recently, exosomes have attracted interest in cardiovascular medicine for their role in cell signaling in a complex system, such as the heart, and in the homeostasis of physiological cardiac functions [33]. It has been demonstrated that stress environments, i.e., hypoxia or inflammation, modulate the content of exosomes and their release within the recipient cells, participating in both improvement and impairment of heart activity [34]. In order to study the cardioprotective properties of exosomes, it is useful to isolate them from cells of several origins: MSCs, induced pluripotent stem cells (iPSCs) and cardiac progenitor cells (CPCs). Arslan et al. found that exosome treatment was able to activate pro-survival pathways by increasing the concentrations of adenosine triphosphate (ATP) and nicotinamide adenine dinucleotide (NADH) and reducing oxidative stress, decreasing infarct size accordingly and enhancing cardiac function [35]. Given the role of exosomes in all the abovementioned conditions, their use may be of potential interest in autism spectrum disorder (ASD).

However, despite these beneficial effects, EVs also play a role in tumor progression [27,36,37]. Three potential therapeutic methods have been proposed: (i) blocking of EV synthesis; (ii) elimination of circulating EVs; (iii) blocking of EV absorption [36]. Several in vivo and in vitro studies evidenced the efficacy of inhibiting EV synthesis in tumor reduction.

## 3. The Capacity of Exosomes to Address ASD-Related Issues

Neurodevelopmental lifelong pathologies defined by problems in social and communication capacity, with the presence of stereotyped and repetitive clusters of behavior and activities, are grouped under the definition of autism spectrum disorder [38]. Speech delay or absence of speech, attention deficit/hyperactive disorder (ADHD) and gastrointestinal (GI) issues are among the co-occurring symptoms associated with ASD [39,40]. ASD is diagnosed in early childhood through the examination of behavior as no specific biological marker has been identified so far [41]. The dramatic increase in ASD prevalence [42] urgently requires biomarker identification and specific treatment options. Even if the exact ASD etiology is still unknown, recent studies indicate interactions between environment and polygenic susceptibility with epigenetic changes as the basis of ASD development [43]. Pro-inflammatory state has been associated with ASD, together with strong immune system abnormalities. Indeed, inflammation in the central nervous system and immune system dysregulation have now been recognized as contributing to the development of ASD [44,45,46]. The molecular and cellular changes underlying ASD have offered the basis for the potential use of stem cells in ASD therapy. Above all, the paracrine effects and immunomodulatory activities of SC have opened the way for their clinical applications in ASD therapy [47]. Whereas it is already recognized that SC option treatment might provide huge benefits for restoration therapy [48], the mechanisms of action of stem cells in restoring disrupted molecular pathways are still to be elucidated. In the same stem cell-related paracrine effect, however, a key role is mediated by the secretome, the complex tool of macromolecules (interleukins, growth factors and extracellular vesicles) secreted by stem cells, responsible for the rescue of damaged tissues [47]. These bioactive factors produced and released by stem cells are able to initiate endogenous repair pathways in the injured organs, providing restorative actions [49]. In addition, through the secretion of these soluble molecules, SC affect several other cell types [50], i.e., immune system cells, providing the rationale for their use in ASD therapy, where the immune system is strongly affected [51]. Probably, the stem cell-mediated paracrine effect is due mainly to the secretome extracellular vesicles, a complex 50–200 nm sub-compartment enriched with proteins/peptides, lipids and short nucleic acids, such as several RNAs [52], by which stem cells are able to transfer bioactive factors to other cells, affecting their functions. As described in the previous paragraph, among the extracellular vesicles secreted by stem cells, exosomes are gaining great attention as they could be the mediators of many of the stem cell-associated therapeutic activities [53]. Through the delivery of exosome cargo (RNA and proteins) to recipient cells, several ASD damaged molecular pathways could be repaired. Indeed, a study demonstrated that MSC-derived exosomes were able to modulate macrophage polarization toward the M2 phenotype [54,55]. Of note, in ASD, peripheral blood mononuclear cell (PBMC)-derived macrophages show dysregulation in an in vitro cellular model [56]. In addition, exosomes exert anti-inflammatory activities in neurological diseases, likely due to stimulation of anti-inflammatory cytokines production [57]. In this way, their application in resolving the strong ASD-associated pro-inflammatory state could be desirable. Exosomes are also able to regulate immune system cells. It has been demonstrated that exosomes extracted from MSCs are able to inhibit IL-17-marked harmful T cells and to induce IL-10-marked regulatory cells in an in vitro model of chronic GVHD [58]. Furthermore, bone marrow-extracted MSC-derived exosomes showed a capacity to block the release of pro-inflammatory molecules TNF-α and IL-1β, and to increase the level of anti-inflammatory molecule TGF-β on in vitro PBMCs [59]. Interestingly, in ASD, GI disorders and common co-occurring conditions have been shown to be associated with elevated inflammatory cytokines, such as TNF-α. Alleviating inflammation in ASD through the use of exosomes could in turn improve GI functioning in those ASD subjects with GI disorders that are associated with inflammation. The same consideration could arise from the fact that pro-inflammatory cytokines are associated with problem behavior, such as irritability and decreased socialization; exosomes could address these issues by resolving the inflammation.

Taken together, these results suggest that exosomes have a key role in immune system modulating, highlighting their potential efficacy in regulating the imbalance of this system in ASD.

Recent findings indicate that neuroinflammation is involved in ASD pathogenesis, as a result of increased pro-inflammatory cytokine levels and microglia/astrocyte aberrant responses in the autistic brain, as well as in autism animal models [60]. Exosomes extracted from human umbilical cord MSCs are able to alleviate brain neuroinflammation by regulating the activation of microglia of Alzheimer’s disease in mice [61]. The intranasal delivery of human Wharton’s jelly MSC-derived exosomes in rat pups with experimental perinatal brain damage showed reduced microglia-mediated neuroinflammation [62].

Interestingly, exosomes could also be used as carriers for the efficient delivery of specific drugs or molecules across the blood–brain barrier (BBB) or other cellular membranes. Curcumin-primed exosomes showed the capacity to counteract in vitro neuronal cell death and to alleviate in vivo the symptoms of Alzheimer’s disease through inhibition of the phosphorylation of the Tau protein and activation of the AKT/GSK-3β pathway [63]. Hence, exosomes could be engineered to modulate abnormal immune functions. Treating the donor cells with several interleukins, such as IL-4 and IL-10, the function and composition of exosomes could be changed ad hoc [64], obtaining a useful drug delivery system to address ASD immunological alterations.

Curiously, helminth worms also secrete extracellular vesicles in order to manipulate interaction with the host [65]. Helminthic therapy has been proposed for addressing ASD immune dysregulations [66]. In the light of recent knowledge, exosomes could be the mechanism by which helminths exert anti-inflammatory activity in ASD.

## 4. In Vivo Models and Clinical Trials

So far, exosome research and applications in ASD are still in their infancy. There are still few experimental studies. However, the research already performed indicates promising results.

In an experimental model of Rett syndrome (the *Mecp2* gene-mutated neurodevelopmental female disease that shares several symptoms with ASD [67]), obtained by MECP2-knockdown in human primary neural cell cultures, it has been demonstrated that exosomes extracted from hiPSC-derived neural cultures were able to rescue the decrease in neuronal growth and differentiation, whereas those extracted from MECP2-deficient neuronal h-iPS cells could not [68]. Interestingly, exosome treatment increased synaptogenesis in neural cultures, indicating that their bioactivity could be useful in treating alterations in synaptic density, as seen in ASD [45].

Exosomes have a small size (30–150 nm) and are constituted by a lipid bilayer, characteristics that permit their easy crossing of the BBB [69]. In several murine brain pathologies, including autism, bone marrow MSC-derived exosomes, intranasally delivered, are able to migrate and home in on different targeted brain areas, driven by neuroinflammatory signals [70]. This aspect seems to be similar to the stem cell homing-like activity, in which stem cells are able to move toward the damaged site, driven by the local chemical environment [47]. This property is of particular interest in potential ASD treatment, as autistic children show altered integrity of the BBB [71]. Therapeutic exosomes could be intravenously administered in ASD subjects, so the nanoparticles move toward the higher brain center through the permeable BBB and exert their effects in ameliorating brain neuroinflammation in situ. In addition, brain connectivity and synaptic density have been demonstrated to be disrupted in ASD subjects. Interestingly, it has been demonstrated that cellular therapy in ASD was able to increase brain frontal, temporal and subcortical white matter connectivity [72]. As a possible mode of action, cellular treatment decreased neural inflammation, in this way promoting the development of white matter connectivity in neural networks and increasing cognitive performance [72]; the potential benefits of cellular therapy could also be due to exosome-mediated effects in reducing inflammation.

Recently, a very important paper demonstrated the efficacy of MSC-derived exosomes in ameliorating autistic-like behaviors in a mouse model of autism. Exosomes were intranasally and intravenously delivered in BTBR mice. Both the administration routes showed efficient homing of the exosomes in the brain after passage of the BBB [73]. Treated BTBR mice showed increased social interaction and decreased repetitive behaviors after three weeks post-exosome treatment; ultrasonic vocalization and maternal pup retrieval were also improved in respect to saline-treated BTBR mice and control wild type C57BL mice [73]. To note, the same research group previously performed MSC (alone) transplantation in BTBR mice, demonstrating amelioration in autistic behaviors in cell-treated BTBR mice [74]. Furthermore, even if detailed molecular characterization of exosomes needs to be performed, the last study suggests that the effective mechanism of action of MSCs in ASD could be due to their secreted exosomes, as postulated in the previous paragraph.

On the other hand, ASD pathophysiology remains to be fully discovered. It has been demonstrated that exosomes derived from the serum of ASD children were able to stimulate cultured human microglial cells to release more pro-inflammatory cytokine IL-1β compared to the exosomes derived from the sera of normal developing children [75]. This latter result indicates a possible method of cellular communication by which neuroinflammation is triggered in the pathophysiology of ASD, but it also suggests a potential target (the pro-inflammatory cargo exosome) in ASD management.

## 5. Conclusions

Exosomes could represent a novel therapeutic tool. The possibility of modulating and/or priming their cargo component in an ad hoc manner could confer powerful uses for this molecular technology. However, this could represent a difficult step as the loading could damage the structure and functionality of the exosome membrane. Before complete application in clinical settings, further studies are required in order to better define the mechanism of action. For example, the precise molecular characterization of MSC-derived exosome cargo is desirable as the molecular content is variable based on the origin of the MSC [76]. Another limitation is the yield of exosome production per cell. Optimal therapeutic doses need to be further evaluated in order to establish a standard protocol for exosome extraction and purification. Indeed, as potentially cell-free drugs, exosomes must accord to the rules of pharmacology: dose, administration route, repeated treatments, biodistribution and pharmacokinetics are to be determined [77]. Until now, it appears that intranasal administration of exosomes offers few side effects. Eventually, adverse effects could be related to the manufacturing, ex vivo/in vitro loading and preparation of exosomes as the composition of the external layer of the lipid membrane could be altered, triggering toxic or immune reactions [78]. Even if MSCs are the optimal source for exosome extraction, other sources could be used. For example, it has been reported that bovine milk and crushed grapes could be interesting alternative sources [78]. The choice between autologous or donor-based exosome extraction should show the same outcomes, as exosomes exert potent immunomodulatory activity, not affecting immunogenicity. If ASD patients show some genetic alterations, these changes will not be kept by the autologous exosomes as cell-free materials.

Due to the huge diversity of the spectrum (ranging from Asperger syndrome to classical autism), ASD endophenotypes should be better characterized in order to address exosome therapy.

Finally, potential exosome therapy, as is the case for all types of cellular therapy [79], will have to be approved by regulatory institutions.
